# Hyaluronan and Associated Biomarkers: A Longitudinal Cohort Study in Patients with Obesity Following Gastric Bypass Surgery

**DOI:** 10.1007/s11695-026-08564-x

**Published:** 2026-03-03

**Authors:** David Thalén, Urban Hellman, Jeff Wennerlund, Ulf Gunnarsson, Magnus Sundbom, Karin Strigård

**Affiliations:** 1 Department of Diagnostics and Intervention, Surgery, Umeå University, Umeå, Sweden; 2 Department of Clinical Microbiology, Umeå University, Umeå, Sweden; 3 Department of Public Health and Clinical Medicine, Umeå University, Umeå, Sweden; 4 Department of Surgical Sciences, Uppsala University, Uppsala, Sweden

**Keywords:** Bariatric surgery, Gastric Bypass, Metabolic effects, Obesity, Hyaluronan

## Abstract

**Introduction:**

Roux-en-Y Gastric Bypass (RYGB) is a common treatment option for obesity. After RYGB, loss of both adipose tissue and lean body mass is seen. In this study, we have investigated the dynamic metabolic changes of hyaluronan (HA) and associated biomarkers reflecting the extracellular matrix after RYGB.

**Materials and Methods:**

In this exploratory cohort study, a total of 306 serum samples were collected at 5 different times from 96 RYGB patients, preoperatively until 6 months after surgery, where 44/96 (46%) contributed samples at 6 months. HA and the cell-surface receptor CD44 were studied by enzyme-linked immunosorbent assay (ELISA), while Luminex Multiplex assays were used for MMP-2, MMP-9, TNF-α, IL-1β, IL-6 and IL-10.

**Results:**

Preoperatively, an elevated HA-concentration (> 120 ng/ml) was seen in 39.6% of the study population. From baseline to day of surgery, we found a statistically significant decrease (*p* < 0.05) in HA (Δ-21.4ng/mL [-42.6, -0.27]), CD44 (Δ-26.7ng/mL [-46.4, -6.9]), MMP-2 (Δ-32.4 ng/mL [-41.4 -23.4]) and MMP-9 (Δ-138.2 ng/mL [-188.0, -88.4]), TNF-α(Δ-3.1 pg/mL [-5.4, -0.8]), IL-1β (Δ-14.4 pg/mL [-22.2, -6.6]) and IL-6 (Δ-2.0 pg/mL [-3.1, -0.9]). At one month postoperatively, a subsequent increase was seen. Although the mean concentration of HA was unchanged at 6 months, patients with baseline HA *≥* 120 ng/mL demonstrated a decrease, (Δ-37.1 [-55.8 to -14.7] *p* < 0.01; exploratory analysis)

**Conclusion:**

Although mean HA levels returned to baseline at 6 months, a significant transient decrease was observed immediately postoperatively, and patients with elevated preoperative HA (> 120ng/ml) showed a sustained reduction. The postoperative increase of MMP-2 suggests a continuous remodeling of the extracellular matrix.

**Supplementary Information:**

The online version contains supplementary material available at 10.1007/s11695-026-08564-x.

## Key points

• Pathological hyaluronan serum concentration is common in patients with obesity but improves after gastric bypass surgery.

• Chronic inflammation caused by obesity persists during weight loss.

• The demonstrated increase in matrix metalloproteinase 2 (MMP-2) indicates postoperative remodeling of the extracellular matrix.

## Introduction

Obesity is a global health issue that leads to an increase in morbidity but also in all-cause mortality. Today, metabolic and bariatric surgery (MBS) is commonly provided as an effective procedure for weight loss that also has beneficial secondary effects regarding incidence of hypertension, hyperlipidemia and type II diabetes (T2D) [1, 2]. It has also been shown that bariatric surgery can improve Metabolic Dysfunction-Associated Steatotic Liver Disease (MASLD) [3], formerly known as NASLD/NAFLD (Non-alcoholic Steatotic/Fatty Liver Disease) [4]. Postoperatively, patients experience reduction of excess fat as well as concomitant loss of lean body mass. The determinants of changes in muscle mass and altered muscular aerobic capacity have been characterized [5, 6].

With obesity, adipose tissue quickly expands related to an abundance of nutrient availability. This expansion has been shown to create hypoxia in the adipose tissue, leading to an inflammatory response and increased angiogenesis, which results in a controlled remodeling of the extracellular matrix (ECM). After a longer period of time, this leads to chronic inflammation with excessive ECM protein deposition, and, ultimately fibrosis [7]. These changes are counteracted by hyaluronan (HA).

HA is a glycosaminoglycan which is abundant in the ECM and known to provide resistance to compressive forces by stabilizing the ECM through hydration. However, HA has more complex properties which vary depending on the length of polymer chains of HA and therefore its molecular size [8, 9]. High-molecular-weight HA (HMW-HA) has been shown to be anti-inflammatory, while low-molecular-weight HA (LMW-HA) can induce and up-regulate pro-inflammatory cytokines, such as interleukin (IL)−1Beta, IL-6 and tumor necrosis factor (TNF)-alpha [10–12]. LMW-HA has previously been shown to be elevated in patients with obesity [10], however a recent study comparing T2D and nondiabetic patients with obesity showed that plasma concentrations of HA with a higher molecular weight above 30 to 35 kDa at baseline was within a normal range but increased at 2 years after Roux-en-Y gastric bypass (RYGB) in both groups [13]. It has also been shown that patients with MASLD and fibrosis had significantly higher HA serum concentrations compared to patients with MASLD but no fibrosis [14]. Given the role of HA in ECM remodeling and inflammation, we hypothesized that bariatric surgery might alter HA dynamics in parallel with metabolic changes.

An important cell surface receptor that HA interacts with is CD44. This receptor regulates several biological processes including inflammation but also affects the expression and activation of important matrix metalloproteinases (MMP) [15]. MMP-2 and MMP-9 degrade proteins in ECM in both physiological and pathological settings. In context of obesity and metabolic surgery, these interactions may contribute to ECM remodeling during weight loss, but human data are scarce. Previous research has however suggested that RYGB influences MMP-2 activity by reducing the gene expression of tissue inhibitor of metalloproteinase 2 (TIMP2) [16]. However, another study investigating both MMP-2 and MMP-9 plasma concentrations showed an increase 1 year after surgery for patients, albeit with diagnosed T2D [17]. RYGB is also associated with a decrease in certain inflammatory markers such as IL-6 [18].

The aim of this exploratory study was to investigate the postoperative changes after RYGB assessing HA and associated biomarkers to characterize the metabolism of the ECM as well as inflammation. We hypothesized that RYGB would reduce serum HA and pro-inflammatory cytokines, reflecting a resolution of ECM remodeling during early weight loss.

## Materials and Methods

### Study Population and Study Design

This was an exploratory study where patients planned for RYGB in 2017–2022 were recruited from Region Västerbotten and Region Uppsala, Sweden. Exclusion criteria included previously diagnosed inflammatory diseases, chronic liver disease and/or malignancy, since previous studies have shown elevated concentrations of HA in these patient groups [9, 19, 20]. Patients who were unable to give written informed consent for any reason were also excluded.

Participants were invited to provide samples at five time points, although not all contributed at each point; prior to surgery (preoperative out-patient clinic), the day of surgery and postoperatively at 1, 3, and 6 months. All patients were given strict low-calorie diet recommendations prior to surgery according to current guidelines. As the study population was recruited from a vast area of Sweden and able to provide blood samples from many locations to increase the probability of continuing this longitudinal study, it was not feasible to require nor control that the patient had been fasting and therefore, this was not specified as a requirement. Blood samples were collected in plastic serum sample tubes with silicone coating as clot activator. Samples were stored at least 60 min at room temperature after phlebotomy and then centrifuged at 2000 g for 10 min. After centrifuging, the samples were aliquoted into smaller tubes without any additives and then stored at −80 °C immediately until analysis. Clinical data was collected from the patients’ medical records and from the Scandinavian Obesity Surgery Registry (SOReg), a validated national quality registry with high acquisition rate [21]. Written informed consent was obtained, and the study was approved by the Regional Ethics Review Board (reference number: 2015/367-31), now known as the Swedish Ethical Review Authority. The study is registered in ISRCTN (ISRCTN17263619).

### Biomarkers

To evaluate the concentrations of HA and characterize the remodeling of the ECM, the following biomarkers were studied: HA, CD44, MMP-2 and MMP-9 as well as TNF-α, IL-1β, IL-6, and IL-10.

### Enzyme-Linked Immunosorbent Assay (ELISA)

Serum concentration of HA were determined using the Echelon Hyaluronan Enzyme-Linked Immunosorbent Assay Kit (Echelon Biosciences Inc., UT, USA. RRID: SCR_004169). This competitive ELISA, also known as an inhibition assay, excels in detecting small analytes in complex mixtures such as plasma. Since HA molecules vary in size and have a wide range of molecular weight, beginning at 1 × 10^5^ Dalton (Da), this method was considered the superior for this study. Serum concentration of CD44 were determined using the Invitrogen Human sCD44std ELISA Kit (Thermo Fischer Scientific Inc., MA, USA). This is a sandwich ELISA detecting all circulating CD44 isoforms that are comprised of the standard protein sequence. For both ELISAs, a coefficient of variation (CV%) under 15 was deemed acceptable and samples with a higher CV% were assessed twice. Both analyses were performed according to the manufacturer’s instructions.

### Luminex Multiplex Analysis

We used the Luminex Multiplex Analysis (R&D Systems Inc., MN, USA. RRID: SCR_006140) to assess the concentrations of IL-1β, IL-6, IL-10, TNF-α, MMP-2 and MMP-9. The multiplex was separated into two different assays according to the manufacturer’s recommended dilutions, 1:2 (IL-1β, IL-6, IL-10, TNF-α) and 1:50 (MMP-2 and MMP-9). A CV% under 12 was deemed acceptable; therefore, samples with a higher CV% were assessed twice in the same manner as for the ELISA assays. All analyses were performed according to the manufacturer’s instructions.

#### Statistical Analysis

IBM^®^ SPSS^®^ Statistics 28 (SPSS Inc, Chicago, IL, USA) was used for statistical analysis. Shapiro Wilks test was used to test for normality. Wilcoxon Signed Rank Test was used to compare preoperative samples with samples given at 6 months postoperatively and Hodge-Lehmann was used to estimate median differences. Bivariate correlation analysis was done using Spearman’s Rho. We fitted linear mixed effects model with random intercept for patient ID and unstructured covariance; time modeled as categorical (preoperative, day of surgery, 1, 3, 6 months). Prespecified interaction (sex*time; age*time) were tested. Missingness was explored (Little’s MCAR test), an available case analysis was made and therefore the number of patients included varies accordingly, no imputation was performed. Adjusting for multiple comparisons, Bonferroni correction was applied. Multivariate analysis was performed using median regression. A p-value < 0.05 was considered statistically significant. As no power calculation was performed, this study should be considered exploratory.

## Results

**Fig. 1:**
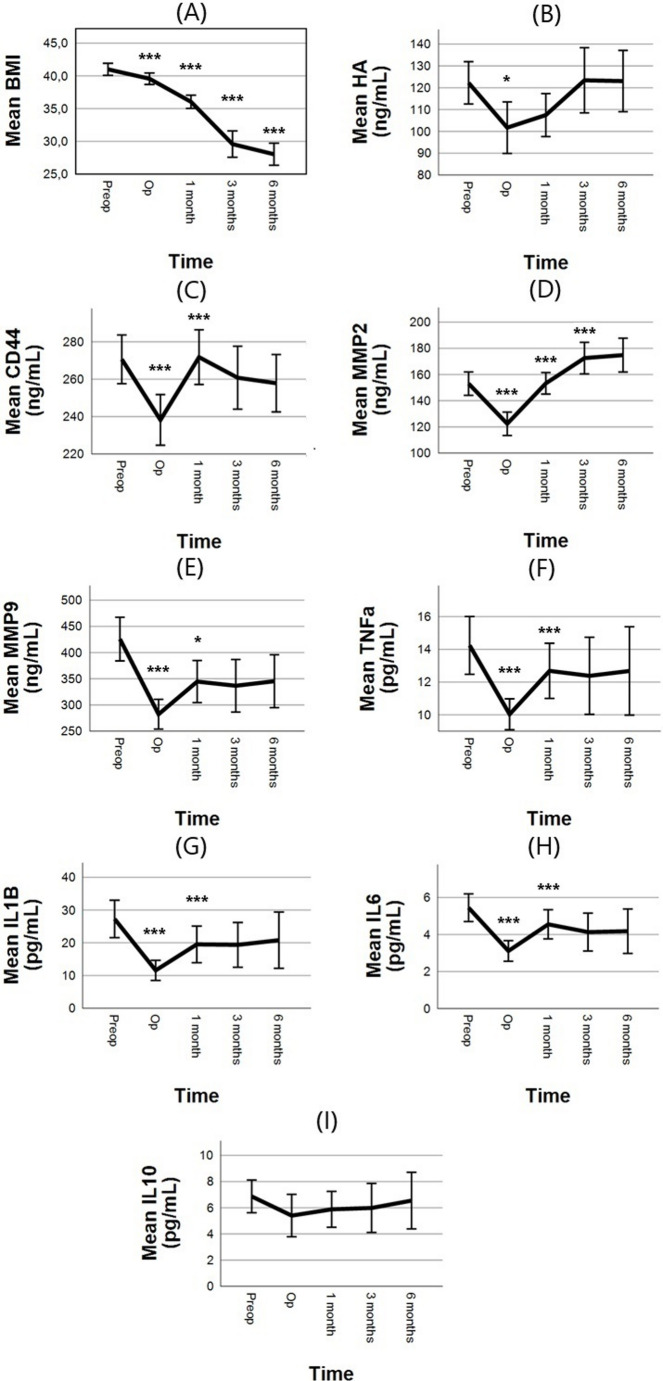
Alterations in clinical variables, p-values are derived from linear mixed effect models. Error bars represent 95% confidence intervals. *N* = 86 preoperatively, 67 day of surgery (Op), 63 at 1 month, 46 at 3 months and 44 at 6). There was a transient early decrease in in all biomarkers except IL-10 at the day of surgery (**A**) BMI = Body Mass Index, (**B**) HA = Hyaluronan, (**C**) CD44, (**D**) MMP2 = Matrix Metalloproteinase2, (**E**) MMP9, (**F**) TNFa = Tumor Necrosis Factor alpha, (**G**) IL = Interleukin-1Beta, (H) IL-6, (**I**) IL-10. P-values are derived from linear mixed effect models. *p-value < 0.05, ***p-value < 0.001

Ninety-six patients (76% females, mean age 41 years (range 25–72) and mean preoperative body mass index (BMI) 41.0 kg/m^2^ (33.4–54.7)) were included in the study. This correlates accurately with the mean age, BMI and gender distribution of Swedish RYGB-patients [22]. Nineteen patients (19.7%) had T2D. During the study period, patients successively lost weight, and had a mean BMI at 6 months of 28.0 (20.8–45.31.8.31, *n* = 37). (Fig. 1A) All patients were non-smokers.

A total of 306 serum samples were collected and analyzed: 86 preoperative samples, 67 samples at the day of surgery, 63 samples 1 month after surgery, 46 samples 3 months after surgery and 44 samples 6 months after surgery (Table 1). The mean number of days between preoperative samples and time of surgery was 113 days. Samples on the day of surgery were collected when the patients had been fasting which was not a requirement for the other measurements. During the study period six patients withdrew their participation, and two patients were lost to follow up. Of the 96 patients included, 10 blood samples were missing at baseline, one patient’s sample could not be analyzed for HA and 2 of the 44 patients that had samples collected at 6 months did not have a preoperative sample and was therefore excluded from the statistical analysis comparing preoperative samples with 6 months and the intra-individual change analysis. As not all participants had given all serum samples, a thorough review of the participants was done, Little’s MCAR test showed that missing data were likely completely missing at random (x^2^(252) = 268.4), *p* = 0.975). When comparing the patients lacking a 6-month blood sample with those who had both a preoperative and 6-month blood sample post-hoc, there was no statistical difference between the groups in regards of age, gender, T2D, preoperative BMI or serum levels of HA and the other biomarkers assessed (See supplementary table).

**Table 1:** Mean concentration of HA and associated biomarkers at every individual time point

Mean (min-max)	Preop	Op	1 month	3 months	6 months
Samples, *n*	86	67	63	46	44
HA (ng/mL)	122.2 (30.1-266.1)	101.7 (43.1-286.8)	107.5. (52.4-223.8)	123.4 (37.3-261.5)	123.1 (57.7-258.0)
CD44 (ng/mL)	270.6 (119.2-414.0)	238.2 (89,1-368.3)	271.8 (110.9-403.4)	260.8 (181.6-490.9)	257.9 (177.8-389.9)
MMP2 (ng/mL)	153.0 (18.8-338.1)	122.4 (37.6-307.2)	153.3 (109.2-295.7)	172.6 (106.9-302.4)	174.8 (110.6-308.2)***
MMP9 (ng/mL)	425.8 (31.5-907.1)	282.3 (63.1-551.7)	344.6 (81.2-676.1)	336.7 (97.4-770.7)	345.4 (102.1-720.1)*
TNFalpha (pg/ML)	14.2 (0.46-36.5)	10.0 (2.0-19.2)	12.7 (2.2-28.9)	12.4 (2.2-37.7)	12.7 (3.29-49.6)
IIIBeta (pg/mL)	27.3 (0.28-94.8)	11.6 (0.28-94.81)	19.5 (0.28-74.9)	19.4 (0.28-93.4)	20.8 (0.28-135.1)
IL6 (pg/mL)	5.4 (0.6-14.2)	3.1 (0.1-14.5)	4.6 (0.3-11.8)	4.1 (0.1-14.2)	4.2 (0.1-21.6)*
IL10 (pg/mL)	6.9 (0.1-21.8)	5.4 (0.1-37.1)	5.9 (0.1-21.4)	6.0 (0.1-24.2)	6.5 (0.1-36.9)

Wilcoxon signed rank test showed a significant change in serum concentration between preoperative samples and 6 months, * = *p *>0.05 *** = *p* >0.001 *HA *Hyaluronan, *CD44* CD44receptor, *MMP *Matrix Metalloproteinase, *TNF *Tumor Necrosis Factdor, *IL *Interleukin

### Hyaluronan

Preoperatively, mean serum concentration of HA was high, 122.2 ng/ml (median 116.8, 30.1–266.1.1.1) with 39.6% (*n* = 38) of the samples exceeding 120 ng/ml, which is above the normal serum concentration of 10–100 ng/ml [23]. When investigating patients with a HA concentration over 120 ng/mL preoperatively, 36.8% (*n* = 14) were male and 31.6% (*n* = 12) had T2D. Bivariate analysis using Spearman’s Rho showed a weak positive correlation between HA and male gender (rs = 0.283. *p* < 0.01, *n* = 85) as well as T2D (rs = 0.277, *p* < 0.01, *n* = 85). Meanwhile, multivariate analysis with median regression adjusting for gender and T2D showed no significant correlation (*p* = 0.7 and 0.3). Interactions of sex*time and age*time did not show any statistical significance. Of the 38 patients with HA concentrations over 120ng/mL preoperatively, 19 had presented a blood sample 6 months after surgery.

Investigating the dynamic changes over time, there was a decrease in mean HA concentration of −19.1 ng/mL (95% CI [−37.5, −0.59], *p* < 0,05, *n* = 67) between preoperative samples and samples at the day of surgery. Following the day of surgery, HA serum concentration increased and were not different at 6 months after the transient early decrease compared to preoperatively (*p* = 1.0) (Fig. 1B) (Table 1).

**Fig. 2:**
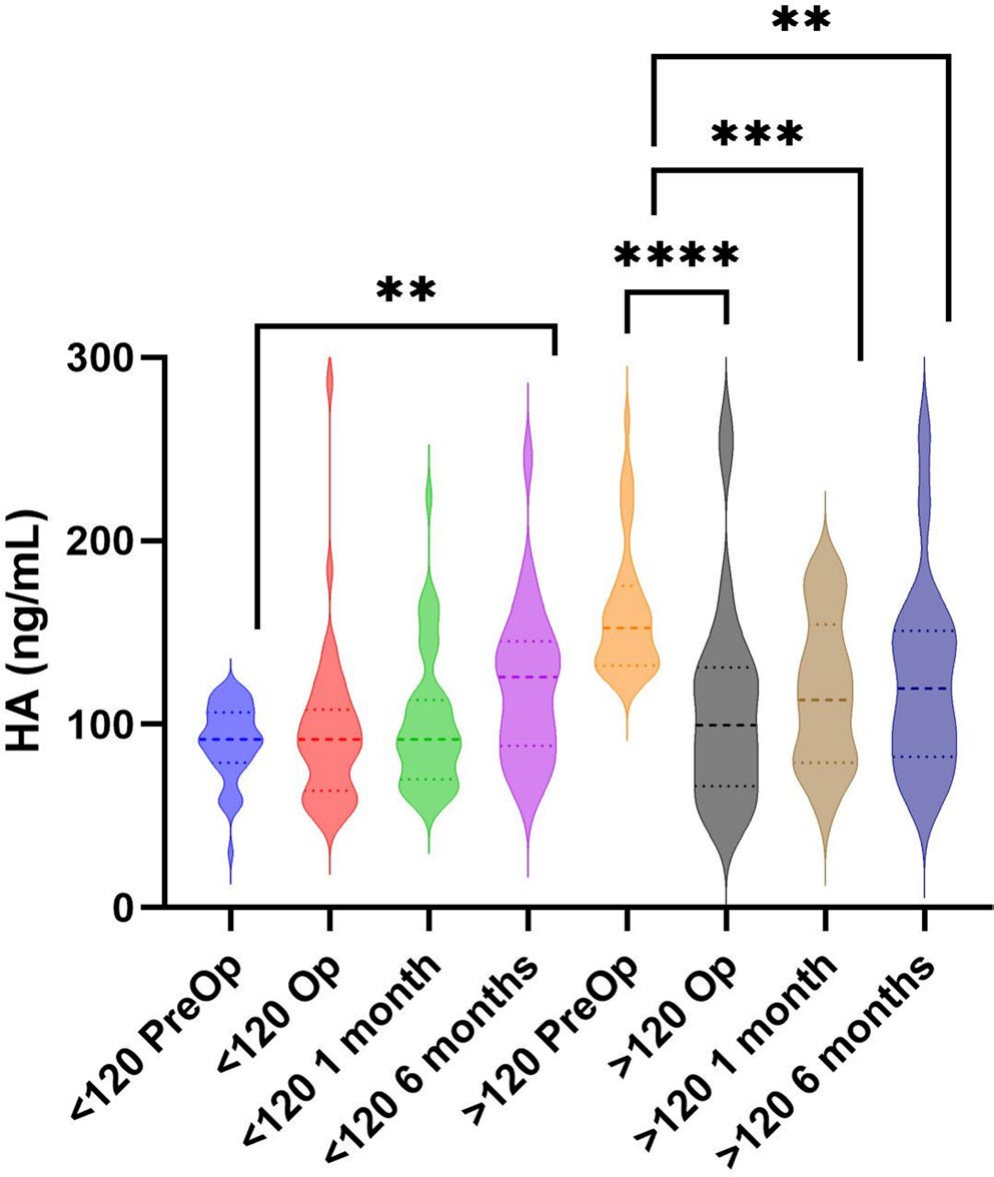
Exploratory post hoc analysis shown in violin plot, separating participants into two groups based on preoperative HA level under/over 120ng/mL respectively, showing differences in HA levels over time and p-values derived from Wilcoxon signed rank test. *N* = 86 preoperatively (< 120 PreOp = 48, > 120 PreOp = 38), 42 at 6 months (< 120 6 months = 23, >120 6 months = 19)., * = *p* < 0.01 ** = *p* < 0.001

A post hoc analysis creating two groups according to HA serum concentration was made as demonstrated in Fig. 2, where participants with a HA below 120 ng/mL preoperatively had an increase in mean HA concentration at 6 months (z = 2.6, Δ 22.7 ng/mL, 95% CI [9.1, 49.3], *p* < 0.01, *n* = 23), while decreasing in participants with HA concentration ≥ 120 preoperatively (z = −2.8,Δ −37.1 ng/mL, 95% CI [−55.8, −14.7], *p* < 0.01, *n* = 19). Using curve estimation, correlations between HA and IL-1β (r (36) = 0.11, *p* < 0.046) as well as TNF-α (r (36) = 0.13, *p* < 0.05) in participants with HA concentration over 120 were statistically significant but very weak and likely not clinically meaningful, and this correlation was not significant at 6 months (*p* = 0.27 and *p* = 0.63 respectively). In participants with HA concentration below 120, this correlation was not shown.

### Associated Biomarkers

Regarding the HA associated biomarkers, a decrease in mean concentration between preoperative and the day of surgery samples was seen for all biomarkers, except IL-10 (Fig. 1C-H); CD44 (Δ −25.6 ng/mL, 95% CI [−42.6, −8.6], *p* < 0.001, *n* = 67), both MMP-2 (Δ −30.7 ng/mL, 95% CI [−38.6, −22.8], *p* < 0.001, *n* = 67) and MMP-9 (Δ −134.1 ng/mL, 95% CI [−176.3, −91.8], *p* < 0.001, *n* = 66), TNF-α (Δ −3.5 pg/mL, 95% CI [−5.4, −1.5], *p* < 0.001, *n* = 67) and also IL-1β (Δ −14.5 pg/mL, 95% CI [−21.0, −8.0], *p* < 0.001, *n* = 67) and IL-6 (Δ −2.2 pg/mL, 95% CI [−3.2, −1.2], *p* < 0.001, *n* = 67). Interactions of sex*time and age*time did not show any statistical significance. Subsequently, the same biomarkers increased between day of surgery and 1 month postoperatively (Table 1); CD44 (Δ 23.4 ng/mL, 95% CI [7.5, 39.4], *p* < 0.001, *n* = 63), both MMP-2 (Δ 30.1 ng/mL, 95% CI [19.2, 40.9], *p* < 0.001, *n* = 63) and MMP-9 (Δ 51.8 ng/mL, 95% CI [8.0, 95.5], *p* = 0.01, *n* = 63), TNF-α (Δ 2.5 pg/mL, 95% CI [0.7, 4.3], *p* < 0.001, *n* = 63) and also IL-1β (Δ 7.3 pg/mL, 95% CI [1.8, 12.9], *p* = 0.003, *n* = 63) and IL-6 (Δ 1.5 pg/mL, 95% CI [0.5, 2.5], *p* < 0.001, *n* = 63). Between 1 month and 3 months, only MMP-2 levels continued to increase (Δ 11.4 ng/mL, 95% CI [2.6 to 20.2], *p* < 0.01, *n* = 46). No statistically significant changes were seen between 3 and 6 months after surgery. Compared to preoperatively, 6 month levels of MMP-2 had increased (178.8 vs. 153.0 ng/mL, z = 3.9, Δ 17.1 ng/mL, 95% CI [9.3, 24.0], *p* < 0.001, *n* = 42), while MMP-9 and IL-6 had decreased (MMP-9 425.8 vs. 345.4, z = −2.28, Δ −57 ng/mL, 95% CI [−101, −10], *p* < 0.05, *n* = 42, IL-6 5.4 vs. 4.2, z = −2.0 Δ −0.8 pg/mL, 95% CI [−1.6, −0.1], *p* < 0.05, *n* = 42) (Table 1).

## Discussion

This is the first study using competitive ELISA to quantify HA of a wide range of molecular weight after metabolic and bariatric surgery. A notable proportion of the patients had high serum concentration of HA preoperatively (> 120 ng/ml) which is often seen in patients with inflammatory diseases such as psoriasis arthritis and liver cirrhosis. However, none of the patients included in the analysis were diagnosed with any such disease. Thus, the high levels could be explained by the chronic inflammation associated with obesity [7, 10, 12, 18]. There was also a positive correlation, albeit weak, between HA and TNF-α as well as HA and IL-1β when assessing patients with HA levels over 120 preoperatively, but this significance was lost in 6 months. Interestingly, mean HA concentration did not decrease after surgery, despite an achieved mean weight loss of 13 BMI-units. The current mean BMI at 6 months, 28.0, indicates that chronic inflammation is still present, albeit diminished. Since weight loss can continue for 12 to 18 months after RYGB, we cannot predict if there will be a complete remission of the chronic inflammation at nadir weight.

While none of the recruited participants in this study had any diagnosis of liver chirrosis nor MASLD at the inclusion, previous research has shown that serum concentrations of HA is elevated in patients with diagnosed MASLD with liver fibrosis, where HA concentration correlates with the stage of fibrosis [14]. As patients’ undergoing RYGB in Sweden is not routinely investigated with further imaging such as ultrasonography regarding MASLD, there is a possibility that some of the participants could have MASLD regardless of lacking any overt pathology during surgery. However, patients planned for RYGB are obliged to provide blood samples prior to surgery including a lipid analysis and transaminases and therefore, in our cohort, should have a medical record with a diagnosis of MASLD or information regarding on-going diagnostics.

Andersson et al. recently demonstrated an increase in HA concentration two years after RYGB in patients with obesity and T2D [13]. Likewise, unoperated patients with T2D had higher HA levels than non-diabetic controls, suggesting a relationship to systemic inflammation [24]. In our study, many of the participants who were diagnosed with T2D had a HA concentration over 120 ng/mL preoperatively but not at 6 months, unfortunately not possible to test statistically due to the small sample size. The method used by Andersson et al. could not detect HA with a molecular weight under 30 to 35 kDa. The competitive ELISA used in this study can quantify HA molecules as small as 26 dimers which is equivalent to 21 kDa. Nevertheless, neither of our methods using ELISA can assess the quota of LMW/HMW HA and therefore discussions regarding LMW HA must be seen as speculative. The findings of Andersson et al. suggest an increase in HMW HA after surgery and, therefore, our unchanged serum concentrations of HA at six months could implicate a simultaneous decrease in the amount of LMW HA. This is, however, speculative, as molecular weights were not measured.

Three months after surgery, there was still a significant increase in MMP-2, ultimately showing a higher mean concentration of MMP-2 at 6 months when compared to preoperatively. This coheres with decreased gene expression of TIMP2 that was shown in a previous study investigating TIMP and MMP gene expression before and after RYGB [16]. Furthermore, we noted a decrease in MMP-9 at 6 months which also suggests a change of remodeling of the ECM. Contrary to this, a recent study on patients with obesity and T2D reported a decrease in serum levels of both MMP-2 and MMP-9 one year after RYGB and sleeve gastrectomy [17], possibly due to partial or complete remission of T2D. In our study population, the concentration of IL-6 was lower at 6 months, previously apparent first after four years [18]. However, both our mean preoperative and 6 months levels of IL-6 in our study participants were within normal range.

In our study, blood samples were centrifuged at least 60 min after phlebotomy and then aliquoted and stored immediately after at −80 °C until analyzed. When analyzing inflammatory markers, previous studies have shown that differences in cytokine concentration in serum samples can be dependent on pre-analytical factors such as time and temperature and therefore, current recommendations are to process samples within the nearest hours from phlebotomy and to store samples at low temperatures. The impact of these variables however differs between different biomarkers [25–27]. Other pre-analytical factors that could be of importance such as the experience of the health-care personnel performing the phlebotomy, time of day for retrieving blood samples and freeze-thaw cycles as well as using assays with the same batch number. To minimize the impact of pre-analytical factors, it is also of utmost importance to handle and process samples with consistency, abiding to a standardized study protocol.

The strength of this study is the novelty of investigating HA in both non-diabetic and T2D patients undergoing RYGB, a previously unexplored field where our method could quantify HA molecules as small as 26 dimers. For assessing HA and associated biomarkers, the present study contains a large cohort compared to previous studies in related research areas. There are some limitations of our study that should be considered when interpreting the findings, as the study participants were only fasting when collecting blood samples on the day of surgery, it could affect cytokine levels and interfere with longitudinal comparisons. A previous study showed that plasma concentration of HA was elevated 1.7–13 times baseline levels with a peak value typically 45–90 min after eating [28]. This should be taken into consideration in our findings as fasting was not a requirement when collecting serum samples except for on the day of surgery. As our study population was geographically spread, it was not feasible to require all samples to be retrieved while fasting and could have resulted in a larger loss of serum samples. However, when investigating the dynamic changes over the whole study period, we could demonstrate long-term changes (preoperatively to 6 months after surgery) in several of our biomarkers. Preoperative weight loss, achieved by the strict low-calorie diet, should not explain the actual values as BMI continued to decrease during the study period. The higher percentage of male patients having HA over normal serum concentration levels where the bivariate analysis showed a significant weak positive correlation that was lost in the multivariate analysis could perhaps be explained by differences in body composition, with an accumulation of abdominal visceral fat in men or hormonal differences. Previous studies regarding differences in HA concentration based on gender is scarce, and to our knowledge only Engström-Laurent et al. have previously showed no notable sex differences regarding serum concentration of HA in their study with over 200 serum samples [29]. However, the investigation of differences in serum concentration of HA in gender was not an aim of this study and this assessment would benefit from a larger study population with a more even distribution of gender. This study is limited by the lack of fasting samples at all time points, a substantial loss to follow-up at 6 months (54% of original cohort), and insufficient power to explore subgroup differences by sex or diabetes status. Although our missing samples data were interpreted as missing completely at random, attrition bias cannot be excluded given that only 46% of patients had samples at 6 months. Also, patients lost to follow-up may differ systematically in unmeasured ways, such as metabolic status or inflammatory burden, limiting generalizability. These limitations should be considered when interpreting the findings.

## Conclusion

In this exploratory study we have shown that obesity is associated with high serum concentration of HA and that there seems to be only a momentary decrease in HA and pro-inflammatory biomarkers in the beginning of weight loss before and directly after surgery. Therefore, biomarker levels suggest ongoing low-grade inflammation at 6 months, but causality cannot be inferred. We did however show a decrease in serum concentration of HA in patients with high HA prior to surgery. The demonstrated increase in serum concentration of MMP-2 suggests postoperative remodeling of the ECM, which is still on-going six months after surgery. Extended follow-up studies after nadir weight has been achieved combined with direct measurement of HA molecular weight distribution are essential to better define its prognostic significance and to elucidate its link to chronic inflammation in obesity.

## Supplementary Information

Below is the link to the electronic supplementary material.

Supplementary Material 1

Supplementary Material 2

## Data Availability

Clinical data was collected from the patients’ medical records and from the Scandinavian Obesity surgery Registry (SOReg). Samples collected during the time period are stored in the research facility associated with the researchers at the University Hospital of Umeå. Raw data are not publicly available to preserve individuals’ privacy under the European General Data Protection Regulation.
