# Supplementary material for: Hyaluronan and Associated Biomarkers: A Longitudinal Cohort Study in Patients with Obesity Following Gastric Bypass Surgery
Source: Obes Surg. 2026 Mar 3;36(4):1696–704. doi: 10.1007/s11695-026-08564-x (PMC13083500; doi:10.1007/s11695-026-08564-x)
Supplement: Supplementary file 1 — Supplementary Material 1 [file 11695_2026_8564_MOESM1_ESM.docx]

**SUPPLEMENTARY TABLE FOR ARTICLE “HYALURONAN AND ASSOCIATED BIOMARKERS: A LONGITUDINAL COHORT STUDY IN PATIENTS WITH OBESITY UNDERGOING GASTRIC BYPASS SURGERY”:**

**
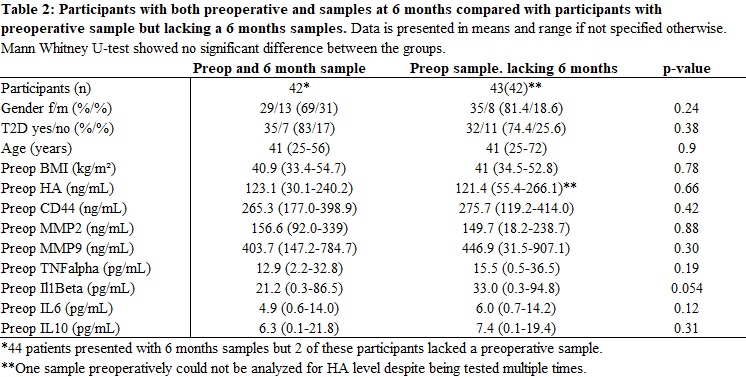
**
