# Supplementary material for: Hyaluronan and Associated Biomarkers: A Longitudinal Cohort Study in Patients with Obesity Following Gastric Bypass Surgery
Source: Obes Surg. 2026 Mar 3;36(4):1696–704. doi: 10.1007/s11695-026-08564-x (PMC13083500; doi:10.1007/s11695-026-08564-x)
Supplement: Supplementary file 2 — Supplementary Material 2 [file 11695_2026_8564_MOESM2_ESM.pptx]

## Slide 1
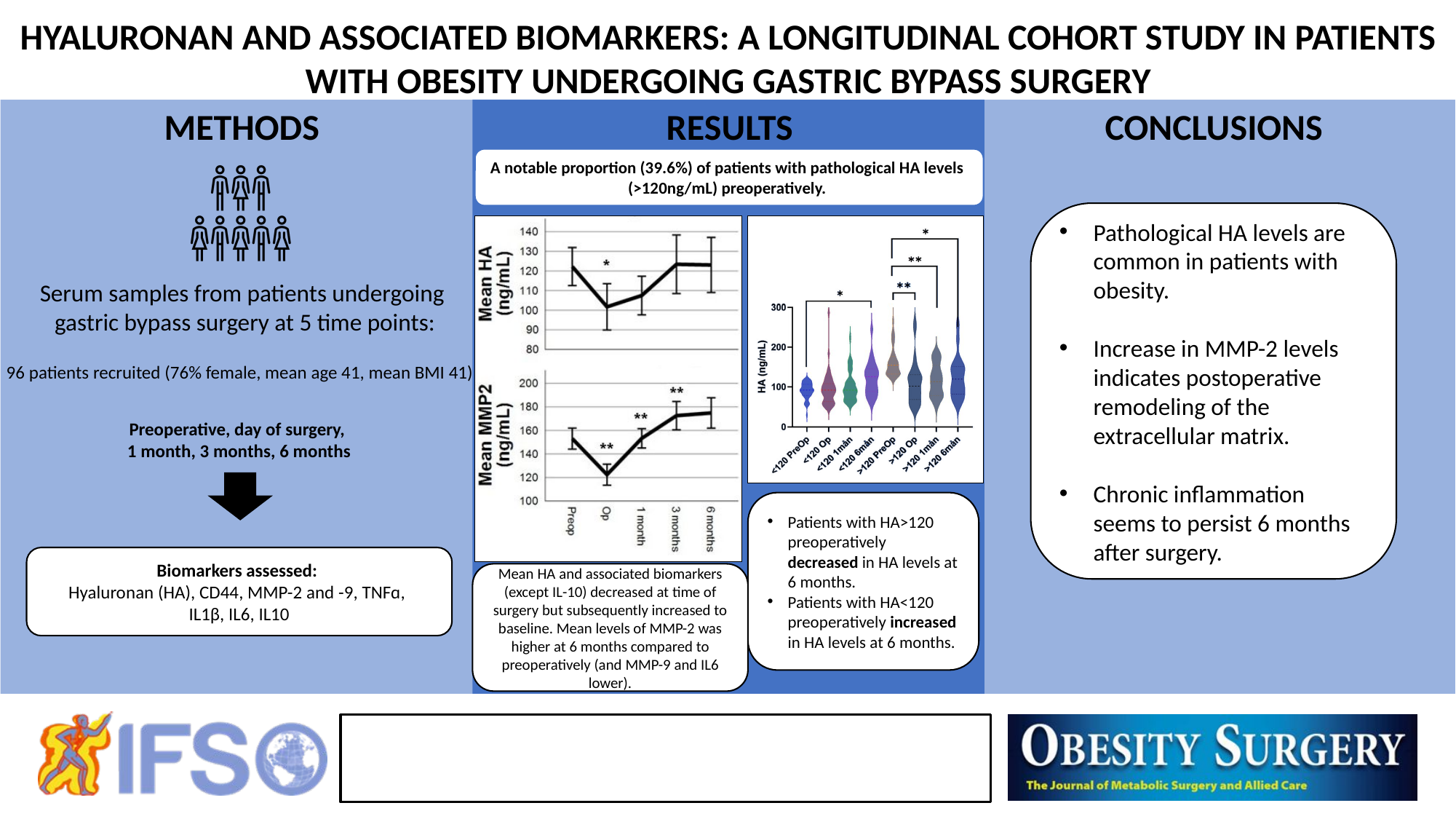

HYALURONAN AND ASSOCIATED BIOMARKERS: A LONGITUDINAL COHORT STUDY IN PATIENTS WITH OBESITY UNDERGOING GASTRIC BYPASS SURGERY
 METHODS
RESULTS
CONCLUSIONS
A notable proportion (39.6%) of patients with pathological HA levels
(>120ng/mL) preoperatively.
Pathological HA levels are common in patients with obesity.
Increase in MMP-2 levels indicates postoperative remodeling of the extracellular matrix.
Chronic inflammation seems to persist 6 months after surgery.
Serum samples from patients undergoing
gastric bypass surgery at 5 time points:
96 patients recruited (76% female, mean age 41, mean BMI 41)
Preoperative, day of surgery,
1 month, 3 months, 6 months
Patients with HA>120 preoperatively decreased in HA levels at 6 months.
Patients with HA<120 preoperatively increased in HA levels at 6 months.
Biomarkers assessed:
Hyaluronan (HA), CD44, MMP-2 and -9, TNFɑ,
 IL1β, IL6, IL10
Mean HA and associated biomarkers (except IL-10) decreased at time of surgery but subsequently increased to baseline. Mean levels of MMP-2 was higher at 6 months compared to preoperatively (and MMP-9 and IL6 lower).
